# Presence of *Alphacoronavirus* in Tree- and Crevice-Dwelling Bats from Portugal

**DOI:** 10.3390/v16030434

**Published:** 2024-03-12

**Authors:** Mahima Hemnani, Priscilla Gomes da Silva, Gertrude Thompson, Patrícia Poeta, Hugo Rebelo, João R. Mesquita

**Affiliations:** 1 School of Medicine and Biomedical Sciences, Porto University, 4050-313 Porto, Portugal; up202110040@edu.icbas.up.pt (M.H.); up202002072@edu.icbas.up.pt (P.G.d.S.); gathompson@icbas.up.pt (G.T.); 2 Epidemiology Research Unit (EPIunit), Institute of Public Health, University of Porto, 4099-002 Porto, Portugal; 3 Laboratório Para a Investigação Integrativa e Translacional em Saúde Populacional (ITR), 4050-313 Porto, Portugal; 4 LEPABE—Laboratory for Process Engineering, Environment, Biotechnology and Energy, Faculty of Engineering, University of Porto, 4099-002 Porto, Portugal; 5 ALiCE—Associate Laboratory in Chemical Engineering, Faculty of Engineering, University of Porto, 4099-002 Porto, Portugal; 6 Biopolis-CIBIO/InBIO Laboratório Associado, Campus de Vairão, 4485-661 Vairão, Portugal; herebelo@fc.ul.pt; 7 Microbiology and Antibiotic Resistance Team (MicroART), Department of Veterinary Sciences, University of Trás-os Montes e Alto Douro, 5000-801 Vila Real, Portugal; ppoeta@utad.pt; 8 Associated Laboratory for Green Chemistry (LAQV), Chemistry Department, Faculty of Science and Technology, University NOVA of Lisbon, 2829-516 Caparica, Portugal; 9 Associate Laboratory for Animal and Veterinary Science (AL4AnimalS), University of Trás-os-Montes and Alto Douro (UTAD), 5000-801 Vila Real, Portugal; 10 Veterinary and Animal Research Centre (CECAV), University of Trás-os-Montes and Alto Douro (UTAD), 5000-801 Vila Real, Portugal; 11 cE3c—Centre for Ecology, Evolution and Environmental Changes & CHANGE—Global Change and Sustainability Institute, Faculty of Sciences, University of Lisbon, 1749-016 Lisbon, Portugal

**Keywords:** coronaviruses, *Alphacoronavirus*, *Pipistrellus pipistrellus*

## Abstract

Coronaviruses (CoVs) are RNA viruses capable of infecting a wide range of hosts, including mammals and birds, and have caused significant epidemics such as the ongoing COVID-19 pandemic. Bats, the second most diverse mammalian order, are hosts for various CoVs due to their unique immune responses and ecological traits. This study investigates CoV prevalence in crevice- and tree-dwelling bats in Portugal, a country with limited prior research on bat CoVs. Using nested RT-PCR and sequencing, we screened 87 stool samples from bats, identifying one sample (1.15%) that was positive for *Alphacoronavirus*, belonging to *Pipistrellus pipistrellus*. Phylogenetic analysis revealed close genetic relationships with *Alphacoronavirus* strains from the same bat species in Europe. The low prevalence suggests habitat-specific differences in viral transmission, with cave-dwelling bats exhibiting higher CoV prevalence due to population density and behaviour. These findings underscore the necessity for sustained surveillance efforts aimed at comprehending CoV dynamics within bat populations, especially concerning the risk of spillover events and viral evolution. Vital to this understanding is the monitoring of bat migration patterns, which serves as a crucial tool for elucidating CoV ecology and epidemiology. Such efforts are essential for ongoing research endeavours aimed at mitigating the potential for future zoonotic disease outbreaks.

## 1. Introduction

Coronavirus (CoVs) are members of the Coronaviridae family, which form a monophyletic group within the Nidovirales order, and are considered the largest known RNA viruses [1]. They possess an enveloped structure, enabling them to infect a broad spectrum of hosts, such as mammals, birds, amphibians, and teleost fish [2]. This versatility leads to a variety of clinical presentations, ranging from asymptomatic infections to severe, often fatal diseases. [3]. The Coronaviridae family comprises three known subfamilies: Letovirinae, which infects frogs; Pitovirinae, which primarily infects fish; and Orthocoronavirinae, responsible for infecting mammals (such as bats) and birds [4,5]. Members of the Orthocoronavirinae subfamily were implicated in the SARS and MERS epidemics, as well as the COVID-19 pandemic, and other several pathogens of clinical, veterinary, and economic importance [6,7,8].

The Orthocoronavirinae primarily target epithelial cells, so are associated with gastrointestinal (faecal–oral route) and respiratory (through contaminated surfaces or objects and respiratory aerosols/droplets) infections [9]. Orthocoronaviruses are divided into four genera: Alpha-, Beta- (which infects mammals), Gamma-, and Delta-Covs (which mainly infects birds) [10,11,12].

Bats (Chiroptera order) are taxonomically diverse, and the second most diverse group among mammals, representing approximately 20% of mammal diversity [13]. They are distributed globally, using a vast array of roosts like rock crevices, caves, and trees, and in urban areas they are frequently found in barns, houses, cellars, and bridges [14]. These roosting sites play pivotal roles in various aspects of bat life. They serve as essential locations for mating rituals, providing safe environments for hibernation during colder seasons, and acting as nurseries for rearing young bats [15,16]. Moreover, these roosts facilitate social interactions among bat populations, allowing for communication and behavioural exchanges critical for their survival [17]. Furthermore, these roosts offer vital protection from harsh weather conditions, such as extreme temperatures and precipitation, as well as from potential predators that pose threats to bat populations [16].

Bats have gained attention for their remarkable propensity to host a vast array of viral species, making them as one of the mammalian groups hosts with the highest viral diversities [8]. This intriguing phenomenon is thought to be intricately linked to a combination of ecological and evolutionary traits unique to bats [13,18]. Understanding the interplay between these ecological and evolutionary factors is essential for unravelling the complex dynamics of viral diversity in bat populations [19]. This underscores the importance of conducting viral research in bats, as it has significant implications not only for public health but also for wildlife conservation and our deeper comprehension of viral evolution [19].

Viral diversity in bats has been explained by a number of factor including bats’ altered immune function that regulates inflammatory genes [20], which can protect them from the development of infectious pathologies [21]. The process of mounting an immune response characterized by excessive inflammation can have deleterious consequences for vertebrates. Such an overactivation of the immune system not only compromises the individual’s health but also imposes a substantial energy cost. While inflammation is a critical component of the immune response, its excessive and prolonged activation can impose significant physiological costs on vertebrates, potentially compromising vital functions such as the growth, reproduction, and maintenance of homeostasis [19]. Therefore, it has been hypothesized that through a series of mechanisms associated with bats’ flying abilities they may be able to tolerate viral infection and replication without the excessive inflammation [22,23]. This flight might produce a fever-like response characterized by elevated metabolism and core body temperature (>38 °C), allowing them to survive viral infections [13]. It is proposed that this tolerance makes infections less virulent toward their natural hosts than novel viruses [24]. Their ability to fly also gives them more mobility than the majority of other mammals, which also enhances the potential for viruses to spread rapidly and widely between bat populations. This, associated with their social organisations, forming large colonies with many individuals in caves, for example, also contributes to the maintenance of viruses in the population [25].

Bats are known to harbour a wide diversity of Alpha and Beta-CoVs. Bats are found to be hosts of at least 30 different CoVs with complete genome sequences available, and many more considering those without whole-genome sequences available [25]. In Europe, to date, there have been 26 studies that have evaluated the presence of CoVs in bats: six studies in Italy, four in Germany, two in the Netherlands, two in Ukraine and one in each of the following countries: Belgium, Bulgaria, Denmark, Finland, France, Hungary, Luxembourg, Romania, Slovenia, Spain, Sweden, Poland, United Kingdom, and Portugal [26,27,28,29,30,31,32,33,34,35,36,37,38,39,40,41,42,43,44,45,46,47,48].

In Portugal, research into the presence of CoVs in bats remains limited, with only one study conducted [49]. This investigation targeted a diverse array of bat roosts such as caves and large buildings across the country, providing an understanding of the distribution and prevalence of CoVs among cave-dwelling bats in Portugal. This approach allowed us to explore the potential reservoirs of CoVs in different ecological settings, shedding light on the complex interactions between bats and their environments. The present study aims to shed light on potential disparities in viral occurrence and diversity among bat populations inhabiting low density bat populations, namely fissure-dwelling settings such as crevices and trees.

## 2. Materials and Methods

### 2.1. Sampling Location

**Figure 1:**
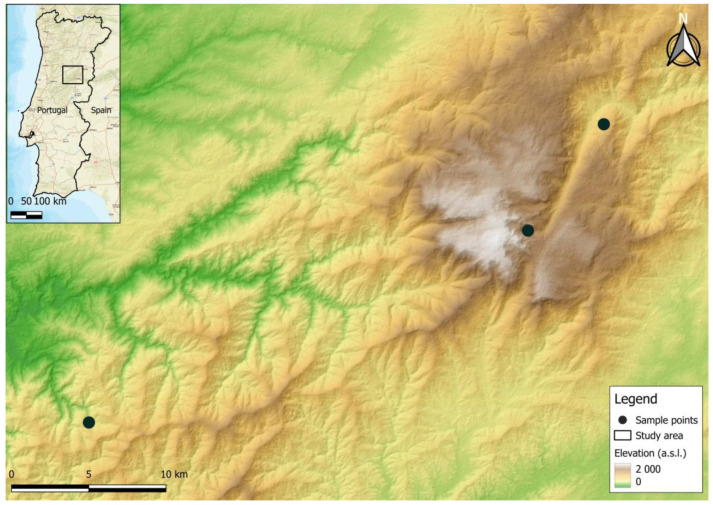
Selected bat sampling locations in Portugal used in this study.

Bat sampling was carried out during June 2023 at three protected landscape areas in the centre of Portugal, in the municipalities of Serra da Estrela (*n* = 2) and Serra do Açor (*n* = 1) (Figure 1). Captures were carried out from dusk till dawn for a total of 3 nights, using steel-framed mist nets.

Captured bats were kept in individual cotton bags until they were processed, and their sex, age and weight were assessed. The captured bats were handled with meticulous care to prioritize their welfare throughout every stage of the procedure. Special attention was given to ensure their safety and minimize any potential stress or discomfort. Additionally, morphological identification was conducted using widely recognized taxonomic keys and guides commonly utilized in bat research. This approach helped to accurately classify the captured individuals based on their physical characteristics, ensuring precise identification and reliable data collection for subsequent analysis. By adhering to established protocols and standards for bat handling and identification, we aimed to uphold the highest standards of animal welfare and scientific rigor in our study [50]. The faeces that were shed during the collection procedure were collected, resulting in a total of 87 stool samples from bats belonging to 7 genera and 13 species. Details of the samples collected can be found in Table 1.

After the capture sessions concluded, great care was taken to swiftly release the bats back into their native environment. This critical step was essential to minimize any potential disruption to their natural behaviours and to ensure the integrity of the research population. By returning the bats to their familiar surroundings without delay, we aimed to mitigate stress and disturbance, allowing them to resume their normal activities without undue interference. This approach not only safeguards the welfare of the bats but also maintains the ecological balance of their habitat, fostering conditions conducive to accurate and reliable research outcomes. All actions pertaining to the capture and handling of bats were executed in strict accordance with the permits provided by the Instituto da Conservação da Natureza e Florestas, guaranteeing full adherence to the rules and recommendations established by the conservation authority (licence number: l 274/2023/CAPT).

### 2.2. Screening for Coronaviruses

The samples were preserved at a temperature of −20 °C until they were ready for further handling. To prepare them, they were mixed thoroughly by vortexing in 500 µL of PBS with a pH of 7.2. The RNA was extracted from the faecal mixture using the QIAamp viral mini kit (Qiagen, Hilden, Germany, reference number: 133226410), following the manufacturer’s instructions and utilizing 140 µL of the clarified supernatants obtained after centrifugation at 1400× *g* for 2 min. The resulting RNA was subsequently stored at −80 °C for future processing.

The extracted RNA was tested for CoVs by employing a broad-spectrum pan-CoV nested RT-PCR assay, which focused on the conserved section of the RNA-dependent RNA polymerase (RdRp) and resulted in a final product measuring 440 bp [51]. The first and second rounds of the PCRs, as well as electrophoresis, were carried out as described in [49,52].

Our approach for the detection and characterisation of CoVs has resourced to a partial RdRp region with primers described by [51]. The sensitivity of the nested pan-CoV primers had been previously assessed by comparing them with other pan-CoV approaches [51]. This thorough assessment determined the efficacy of the primers in the broad spectrum detection of CoVs and ultimately reached the conclusion that the chances of detecting both recognized and unidentified CoVs in a wide range of sample sources were increased [51]. It has been documented that utilizing a small segment of the RdRp from CoVs is sufficient for determining taxonomic classifications even at the subgenus level [53]. This accuracy in classification is similar to what can be achieved using complete genome sequences [53].

### 2.3. Sanger Sequencing and Phylogenetic Analysis

The next step involved isolating the positive amplicon using the GRS PCR Purification Kit (Grisp, Porto, Portugal, reference number: GK64.0100). Subsequently, bidirectional sequencing using the specific primers [51] for the target gene was carried out through Sanger sequencing. The sequence was then aligned using alignment clustals with the software package BioEdit Sequence Alignment Editor v7.1.9, version 2.1 (Ibis Biosciences, Carlsbad, CA, USA) and compared with the sequences available in the NCBI nucleotide database (GenBank, Carlsbad, CA, USA) (https://blast.ncbi.nlm.nih.gov/Blast.cgi, accessed on 11 November 2023). The obtained sequence was included for phylogenetic analysis and submitted to GenBank under the accession number OR625571.

This sequence was aligned with other 55 reference strains representing the four CoV genera (Alpha-, Beta-, Gamma-, and Deltacoronavirus), all sourced from GenBank. The alignment process was conducted using MEGA X software [53]. The MEGA X software’s model selection function was employed to choose the model with the lowest Bayesian information criterion (BIC) score [54]. This selection was based on the maximum likelihood method and utilized the general time reversible model with a discrete Gamma distribution, assuming evolutionarily invariable sites. This was followed by 1000 bootstrap replicates. The final step involved editing the alignment with the Interactive Tree of Life (iTOL) platform [55].

## 3. Results

In this study, from the collection of 87 stool samples from bats from three sites, only one sample (1.15%; 95% confidence interval [CI]: 0.03–6.24) exhibited amplicons of the expected size for CoV using the pan-CoV-nested RT-PCR. It was then further analysed through bidirectional sequencing and nucleotide BLAST analysis. The bat testing positive for CoV belonged to the species *Pipistrellus pipistrellus* and was collected during the first day at Serra da Estrela.

**Figure 2:**
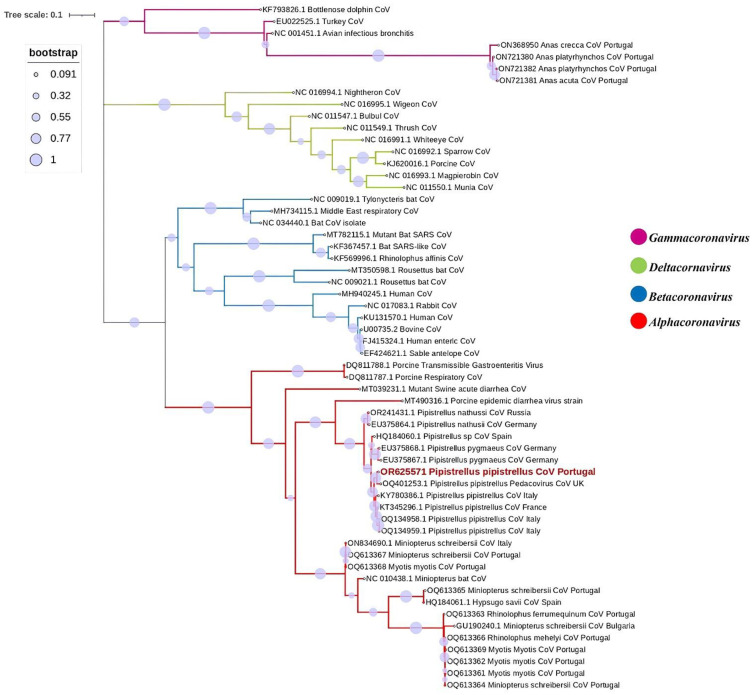
Phylogenetic tree constructed for the Gamma-, Delta-, Beta- and Alphacoronavirus, using 57 reference strains and the 1 strain identified in this study. Phylogenetic analysis was based on a 406 nt partial region of the RdRp. The tree was constructed using MEGA X and the maximum likelihood based on the GTR + G model, and 1000 bootstraps were replicated. The sample from this study is indicated in red, with the description of sample number, GenBank accession number, and host bat species.

The retrieved CoV sequence was assigned the accession number OR625571. BLAST analyses provided highest hits to sequences from *Alphacoronavirus*. Additional characterisation through BLAST showed that the obtained sequence displayed highest similarities to CoVs retrieved from other *Pipistrellus pipistrellus*. The highest identities were revealed as the previously identified *Alphacoronavirus* in the United Kingdom (West Sussex), recorded under the accession number OQ401253, with a percentage identity of 98.04%, followed by an *Alphacoronavirus* sequence previously identified in France under the accession number KT345296, with a percentage identity of 96.93%, and another three *Alphacoronavirus* sequences had been previously identified in Italy under the accession numbers KY780386, OQ134959, and OQ134958, with percentage identities of 96.39–96.67%. Phylogenetic analysis was subsequently conducted using the acquired CoV sequence alongside 57 reference strains. The analysis confirmed its placement within the *Alphacoronavirus* genus, sugenus *Pedacovirus*, as illustrated in Figure 2.

## 4. Discussion

In this research, our primary objective focused on the investigation of the presence and characterisation of CoVs specifically within the crevice- and tree-dwelling bat populations inhabiting Portugal. This study stands as a milestone, marking the second recorded instance of CoVs detected in bats in Portugal. Notably, it represents the pioneering documentation of CoVs in non-cave-dwelling bat species within the region. By delving into the viral ecology and diversity inherent to these bat populations, our research provides crucial insights into the intricate dynamics of CoV transmission and the potential reservoirs of these viruses within bat communities. Through our findings, we aim to contribute significantly to the collective understanding of zoonotic disease dynamics, ultimately aiding in the formulation of effective strategies for disease surveillance, prevention, and management.

We conducted screening on a total of 87 stool samples using nested RT-PCR followed by sequencing. From this screening only one sample showed to be positive for Alpha-CoV (1.15%; 95% confidence interval [CI]: 0.03–6.24), which is low compared to other studies that reported prevalence rates usually ranging from 3% to 17% [26,41,56,57]. Unexpectedly, our prior investigation into CoV excretion in Portugal revealed an occurrence of 8.87%, indicating a higher prevalence of CoVs in cave-dwelling bats than crevice- and tree-dwelling bats [49]. This could be because of different habitat characteristics, variations in bat species behaviour, proximity to potential virus reservoirs, and fluctuations in bat population density.

These factors can potentially influence the likelihood of detecting CoV in bat populations. Even if the study had opted for a country-wide and multi-year surveillance, the prevalence rate might not necessarily increase. This is because the effectiveness of surveillance efforts depends on various factors beyond the scope of the study design, such as the choice of sampling locations. Conducting surveillance in different habitats, such as caves or other bat habitats, may yield different results due to variations in environmental conditions and bat species composition, and more importantly, animal densities. The CoV strain identified in the bat in our study exhibited close genetic relationships with *Alphacoronavirus* strains previously isolated from the same bat species, *P. pipistrellus*, in the United Kingdom (West Sussex) [40], more specifically, from the *Pedacovirus* subgenus, and it also clustered with other strains previously isolated from *P. pipistrellus* in France and Italy [42,43]. The phylogenetic tree based on the partial RdRp gene confirmed that our sequence clustered with other members of the *Alphacoronavirus* genus retrieved from *P. pipistrelus* with strong support, as indicated by >90% bootstrap value. Moreover, the retrieved *Pedacovirus* sequence also clustered with sequences found in other *Pipistrelus* spp. throughout Europe, namely in *P. nathusii* and *P. pygmaeus*, suggesting host specificity and raising important questions on viral circulation.

Our phylogenetic tree analysed several bat Alpha-CoV sequences from GenBank and the Alpha-CoV found in our study. Surprisingly, all strains were retrieved from the same bat genus (*Pipistrellus*), and in most cases, from the same species (*Pipistrellus pipistrellus*), forming the same monophyletic branch. This clustering strongly implies that closely related viruses are prevalent within the same host species, even when geographically separated. This phenomenon is corroborated by previous research findings, indicating a compelling pattern of viral association with specific bat hosts irrespective of geographical location [56,58].

In contrast, the research involving cave-dwelling bats revealed that the clustering of bat CoVs was not primarily determined by the species of bat. Instead, it appeared that geographical location played a more significant role in influencing the evolution and dissemination of these viruses [49]. This suggests that environmental factors, such as habitat characteristics and local ecological dynamics, may exert a more significant influence on the genetic diversity and distribution of coronaviruses in cave-dwelling bat populations.

*P. pipistrellus* distribution encompasses a substantial portion of Europe, with confirmed sightings extending as far north as Norway and southern Finland. To the south, the presence of this species has been established in Greece, Cyprus, and Turkey [59]. Beyond the borders of Europe, the species has been observed in northwest Africa, Asia Minor, and across the Middle East to Iran and Afghanistan [59]. In Portugal, this species is widely distributed across the entire national territory and is considered one of the most abundant bat species in the country [50]. These bats, characterized by their preference for dwelling in crevices, commonly seek refuge in various human-made structures, leading them to roost in buildings, warehouses, and architectural relics such as churches and castles, among others. Their adaptation to utilizing human infrastructures for roosting highlights their status as synanthropic species, demonstrating their ability to thrive in close proximity to human populations [60]. This close association with human habitats not only shows the remarkable adaptability of these bats but also the importance of understanding their ecological dynamics in urban environments. However, due to its high levels of adaptability, this species can also utilize crevices in rocks, cliffs, tree cavities, and occasionally, underground shelters during the hibernation season, as well as also seeking shelter beneath tree bark [61].

*P. pipistrellus* are bats that are not known for flying long distances, but recent findings regarding their genetic information indicates a significant degree of genetic exchange within summer colonies in central Europe [62]. This observation implies two distinct possibilities: the occurrence migrations motivated by the search for suitable mates in distant habitats, also propelled by factors such as competition for resources or territory, or the need to establish new breeding grounds [63]. That being said, bats of this species do not have a strong migratory character, since they are usually known to remain confined to their own colonies even if another colony is located 10km away [43]. This also indicates that these viruses may not primarily evolve within a specific bat species, instead, their evolution and spread appear to be significantly influenced by geographical location. This suggests that factors such as environmental conditions, host availability, and interactions with other wildlife species could play pivotal roles in shaping the evolutionary dynamics and spread of these viruses across different regions. Another possible interpretation is that the presence of similar strains in geographically separated areas could be attributed to transmission occurring during the migration of bats. Considering the genetic divergence observed between the sampled strain and others, it is likely that direct transmission has not occurred, but rather, it would likely entail the involvement of intermediate strains that have not been included in the sampled data [56].

All these findings combined suggest the existence of *Pedacovirus*-like Alpha-CoV strains specific to bats species (*P. pipistrellus*), although with low occurrence, underscores the significance of understanding viral diversity within bat populations. While the occurrence of these strains may be relatively low, their presence highlights the potential for novel viral variants to emerge and circulate among bat species [64]. Moreover, the observation that this pattern may extend to synanthropic bat species, which inhabit a wide range of roosting habitats, including natural environments and human-built structures like roofs and windows, is of considerable importance. This suggests that human activities and urbanisation may play a role in shaping the viral ecology of bats, potentially facilitating spillover events and the transmission of bat-borne viruses to other wildlife and human populations [18]. These findings emphasize the need for continued surveillance and research efforts to monitor viral diversity in bat populations and assess the associated risks to public health and wildlife conservation.

Bats play crucial roles as ecosystem service providers, making significant contributions to biodiversity conservation and ecosystem health. Their diverse dietary preferences and foraging behaviours make them effective suppressors of agro-forestry pests and disease vectors. By preying on insects such as moths, beetles, and mosquitoes, bats help regulate insect populations, thereby reducing agricultural damage and the spread of vector-borne diseases [65]. Additionally, bats’ role as pollinators for various plant species contributes to the maintenance of plant diversity and ecosystem stability. Thus, recognizing the ecological importance of bats highlights the need for their conservation and the preservation of their habitats to ensure the continued provision of these valuable ecosystem services [66].

Human-induced alterations, such as deforestation, habitat fragmentation, and land conversion for agriculture and urban development, can exert profoundly adverse effects on the transmission of infectious diseases from wild animals to both livestock and humans [25] and favour the emergence of infectious diseases. The faecal–oral route has also been described in CoVs from other animals such as feline CoVs (FCoV), canine CoVs, and swine CoVs (SADS-CoV)—all of which are classified as Alpha-CoV, highlighting the potential for similar transmission mechanisms across diverse animal taxa [49] Both Alpha- and Beta-CoVs have high detection rates in bat stool samples, suggesting gastrointestinal replication and that these animals’ excretions are a major environmental source for the shedding of CoVs in spillover events [67,68]. A previous study also confirmed the gastrointestinal replication by not finding evidence of CoVs in the caves where bats and bat excretion was found [49]. Understanding the faecal–oral route in these CoVs not only provides insights into their pathogenesis but also underscores the importance of comprehensive surveillance and control measures to mitigate the risk of interspecies transmission and potential outbreaks.

## 5. Conclusions

This study provides unique results for the prevalence and dynamics of CoVs in Portuguese bats. Before this study, our understanding of bat CoVs in Portugal was limited to studies on cave-dwelling species. Our findings now reveal that CoVs are also present, although at a much lower frequency, in crevice- and tree-dwelling bats. Data here presented also strongly reinforce a monophyletic clade within CoVs circulating in the *Pipistrellus* spp. bats of Europe. This clade exhibits close genetic relatedness to other Pedacoviruses, known to cause pathogenic effects in various animal species, suggesting the potential role of bats when considering CoVs species barrier crossing. This suggests that these bats may serve as important reservoirs or vectors for coronaviruses, thereby contributing to the emergence and dissemination of novel viral strains with zoonotic potential. Additionally, the genetic disparity observed between *Pipistrellus*-associated CoVs and other Alpha-CoVs found in bats within Portugal shows a significant phylogenetic distinction. This genetic differentiation strongly implies a physical segregation between *Pipistrellus* spp. bats and cave-dwelling bat species. Such genetic and ecological partitioning highlights the complex dynamics of bat communities and their respective roles in harbouring and transmitting coronaviruses, emphasizing the need for further research to elucidate the mechanisms driving these patterns of viral diversity and distribution. It is of paramount importance to maintain ongoing monitoring of CoVs in bats to establish a foundational dataset for future surveillance. Furthermore, monitoring the migratory behaviours of bats is essential in enhancing our comprehension of CoV evolution, ecology, and other bat-associated viruses and their epidemiology.

## Figures and Tables

**Table 1:** Bat species, number of individuals, and number of stool samples according to collection site.

Location	Species	Number of Individuals Found in Each Location	Stool Samples Collected from Each Species
**Serra da Estrela, day one**	*Pipistrellus pipistrellus*	11	10
	*Eptesicus serotinus*	2	2
	*Myotis emarginatus*	2	2
	*Nyctalus leisleri*	5	4
	*Barbastella barbastellus*	1	0
	*Nyctalus lasiopterus*	1	0
**Serra da Estrela, day two**	*Eptesicus serotinus/isabellinus*	2	2
	*Myotis daubentonii*	1	1
	*Plecotus austriacus*	1	1
	*Plecotus auritus*	6	6
	*Nyctalus leisleri*	1	1
	*Hypsugo savii*	4	3
	*Pipistrellus pipistrellus*	11	11
	*Myotis mystacinus*	3	3
	*Eptesicus serotinus*	1	0
	*Barbastella barbastellus*	1	1
**Serra do Açor**	*Nyctalus leisleri*	13	12
	*Nyctalus lasiopterus*	10	10
	*Pipistrellus pipistrellus*	2	2
	*Plecotus auritus*	15	13
	*Eptesicus serotinus*	1	1
	*Myotis bechsteinii*	1	1

## Data Availability

Data are contained within the article.
